# Nonmedical prescriber experiences of training and competence to report adverse drug reactions in the UK

**DOI:** 10.1111/jcpt.12756

**Published:** 2018-09-11

**Authors:** Andrew Thompson, Christine Randall, Justine Howard, Catrin Barker, Debbie Bowden, Paul Mooney, Agatha Munyika, Susan Smith, Munir Pirmohamed

**Affiliations:** ^1^ Wolfson Centre for Personalised Medicine, Institute of Translational Medicine University of Liverpool Liverpool UK; ^2^ Pharmacy Department North West Medicines Information Centre Liverpool UK; ^3^ Pharmacy Department Alder Hey Children's NHS Foundation Trust Liverpool UK; ^4^ Liverpool Community Health NHS Trust Liverpool UK; ^5^ Pharmacy Department Royal Liverpool and Broadgreen University Hospitals NHS Trust Liverpool UK; ^6^ Pharmacy Department Mersey Care NHS Foundation Trust Liverpool UK; ^7^ Medicines Information University Hospital Aintree Liverpool UK

**Keywords:** adverse drug reactions, patient safety, pharmacovigilance

## Abstract

**What is known and objective:**

Adverse drug reaction reporting in the UK is lower than expected based on epidemiological data. This study aims to explore (a) nonmedical prescribers’ (NMP) confidence in identifying and reporting ADRs, (b) NMP prescribing habits and engagement with the Yellow Card Scheme (YCS) and (c) NMP desire for future training in the identification and reporting of ADRs.

**Methods:**

A survey was distributed across NMP networks in the north‐west of England using Survey Monkey. Univariate analyses were performed to compare the features of reporters and nonreporters, Kruskal‐Wallis H tests for comparisons within multiple subgroups and Spearman's rank correlation coefficient for response associations between answers to ordered‐category questions.

**Results and discussion:**

A total of 570 responses were available for analysis, an estimated response rate of 20%. Less than half (n = 219; 38.4%) reported submitting a Yellow Card to the YCS, and the majority of those individuals have submitted five or less Yellow Cards; 28 responders reported more than five submissions. Being professionally qualified for more years (linear regression: *B* = 0.30, *P* < 0.0005; 95% CI 1.01 to 1.05) and receiving additional training support about the YCS (chi‐squared: *χ* ^2^ = 14.7, *P* < 0.0005) were associated with an increased likelihood of submitting to the YCS. There was a positive linear relationship between confidence in identifying ADRs and likelihood of reporting to YCS. The most common reason given (n = 261) for never having reported to the YCS was “I have never seen an adverse drug reaction.” Training appears to give NMPs confidence in reporting ADRs, but there seems to be a gap in actually identifying ADRs given the comment that most had never seen an ADR.

**What is new and conclusion:**

Strategies for improving the translation of theoretical knowledge about ADRs into practical skills in identifying ADRs, and subsequently reporting them, will be important for improving pharmacovigilance practice.

## 1 WHAT IS KNOWN AND OBJECTIVE

Adverse drug reactions (ADRs) are common. Epidemiological data from the UK suggest that ADRs are responsible for significant healthcare utilization accounting for 6.5% of hospital admissions.^1^ ADRs are detected throughout the course of drug development, but these usually tend to be the commoner and milder reactions. The more serious ADRs are often not detected until the drug is licensed and used in larger population groups. Furthermore, the pattern of ADRs, even milder reactions, may change once the drug is marketed as its use in a real‐world setting will lead to exposure to a wider group of patients (for example, the elderly, those with concomitant disease, those on interacting drugs and off‐label use).^2^, ^3^, ^4^ Postmarketing surveillance is therefore essential to ensure that the risk‐benefit profile of a drug is monitored throughout its life cycle and to identify any new signals of ADRs as soon as possible.

The Yellow Card Scheme (YCS) was introduced in the UK in 1964 following the thalidomide disaster.^5^ The system is the cornerstone of pharmacovigilance in the UK and is coordinated by the Medicines and Healthcare products Regulatory Agency (MHRA). The YCS relies on spontaneous reports which are used alongside data from postmarketing authorization studies and clinical trials to continuously update prescribing information. Identifying and reporting suspected ADRs to the YCS are considered good practice for all healthcare professionals and are included within the competency frameworks which underpin prescribing practice for medical and nonmedical prescribers.^6^

Nonmedical prescribing was introduced to allow healthcare professionals such as nurses, pharmacists, radiologists and physiotherapists to prescribe independently. Certification is gained through formal training, which generally consists of lectures and peer‐supervised practice, and subsequent assessment. The qualification is recognized and reported by each of the profession‐specific governing bodies (eg Royal College of Nursing). An independent nonmedical prescriber (NMP) theoretically can prescribe from the whole of the British National Formulary, but as with medical prescribers, NMP prescribing is usually dependent upon their training, clinical specialty and competence. Nonmedical prescribing was introduced to help improve flexible team working across the UK NHS, allow patients quicker access to the medicines they need and improve patient care without compromising patient safety.

It is important that NMPs receive training on the identification and reporting of ADRs, initially within their course and subsequently once in clinical practice. A study by Stewart and colleagues showed that NMPs have a positive attitude towards ADR reporting, but many lacked sufficient knowledge to answer factual questions about the YCS.^7^ Here, we aim to add to this knowledge by exploring how confidence in pharmacovigilance influences detection and reporting of ADRs. We have therefore undertaken a question‐based survey of NMPs in north‐west (NW) England. The specific aims of this study were to (a) gain an understanding of NMP confidence in identifying and reporting ADRs, (b) explore NMP prescribing habits and engagement with the YCS and (c) investigate the desire of NMPs for future training in the identification and reporting of ADRs. By exploring current confidence levels in ADR identification and engagement with reporting to the YCS, we can help provide a baseline for future quality targets and potentially identify areas of training need.

## 2 METHODS

### 2.1 Study design and participants

A survey was developed by members of Liverpool Health Partners Yellow Card Working Group and Yellow Card Centre North West. Each complete draft of the survey was tested on blinded NMPs, and feedback was used to refine the questions included and the structure; there were four iterations. The survey was distributed via local and regional NMP leads in the NW of England using SurveyMonkey (https://www.surveymonkey.com/). The regional NMP leads have access to all NMPs that have registered with them, but this might not cover the entire NMP regional cohort. We therefore do not have a precise denominator for the number of individuals who received the survey, but estimates suggest that there are around 3000 NMPs in the NW region. No restrictions were placed on respondents, beyond the need to be a qualified NMP. The survey was opened online on 5 September 2016 and closed 9 weeks later. Whether reminders about the questionnaire were distributed was dependent on the local and regional NMP leads.

### 2.2 Data collection

All of the participants were asked about their profession, qualifications, recent prescribing practice, ADR reporting and desire for future training in identifying and reporting ADRs. The flow of the rest of the questionnaire was then dependent upon the responses provided to certain questions. For example, answering “Yes” to “Have you ever reported an adverse drug reaction on a Yellow Card?” meant that participants were asked “Roughly how many Yellow Cards have you submitted?”.

### 2.3 Statistical analysis

Descriptive statistics were performed on the whole sample (all responders) and on the two different groups (YC reporters and nonreporters). Univariate analyses were performed to compare the features of reporters and nonreporters, using the Wilcoxon signed‐rank test for continuous variables and chi‐square for categorical variables. Comparisons within multiple subgroups, for example within answer patterns to ordered‐category questions, were performed using Kruskal‐Wallis H tests for continuous variables. Response associations between answers to the two ordered‐category questions were determined using Spearman's rank correlation coefficient (ρ). Analyses were performed using SPSS 22 software (IBM Corp. Released in 2013. IBM SPSS Statistics for Windows, Version 22.0., IBM Corp, Armonk, NY, USA). The threshold for statistical significance was set at 0.05.

## 3 RESULTS AND DISCUSSION

### 3.1 Results

Table 1 lists the complete question structure and describes the response pattern to each question. Responses were received from 611 NMPs; 41 incomplete questionnaires were rejected, leaving a total of 570 for analysis.

**Table 1:** Survey questions and responses

Question	Responses
A) What is your healthcare profession?	Community practitioner (district nurse/health visitor) = 120 (21.1%); Nurse =388 (68.1%); Pharmacist =27; (4.7%); Other =35 (6.1%)
B) In which year did you gain your professional qualification?	Median =1996; range =1970−2016
C) At which Institute did you complete your NMP course?	Edge Hill University =38 (6.7%); Liverpool John Moores University =28 (4.9%); Manchester Metropolitan University =74 (13.0%); University of Bolton =140 (24.6%); University of Central Lancashire =68 (11.9%); University of Chester =45 (7.9%); University of Cumbria =54 (9.5%); University of Liverpool =9 (1.6%); University of Manchester =10 (1.8%); University of Salford =71 (12.5%); Other =33 (5.8%)
D) In which year did you complete your NMP course?	Median =2010; range =1989−2016
E) In the past year, how often have you prescribed?	At least once daily =260 (45.6%); At least once monthly =73 (12.8%); At least once weekly =176 (30.9%); Less than monthly =41 (7.2%); Never =16 (2.8%); NA to current role =4 (0.7%)
F) After completing your NMP course, how do you feel the following statement applied to you? “I feel very confident about being able to detect and report adverse drug reactions”	Strongly agree =175 (30.7%); Agree =309 (54.2%); Neither agree nor disagree =67 (11.8%); Disagree =19 (3.3%)
G) On your NMP course, were you informed about the Yellow Card Scheme?	Yes =540 (94.7%); No =3 (0.5%); Not sure =27 (4.7%)
H) How was this information about the Yellow Card Scheme delivered?	Face‐to‐face dedicated session =183 (32.1%); Paper‐based distance learning session =3 (0.5%); Online distance learning session =6 (1.1%); Face‐to‐face delivery as part of another session =319 (56.0%); Online distance learning as part of another session =8 (1.4%); Other =22 (3.9%)
I) Were you required to complete a Yellow Card as part of your NMP training?	Yes =152 (26.7%); No =292 (51.2%); Not sure =126 (22.1%)
J) How confident are you are at identifying adverse drug reactions?	Very confident =52 (9.1%); Confident =380 (66.7%); Neither confident nor not confident =118 (20.7%); Not confident =20 (3.5%)
K) Have you ever reported an adverse drug reaction on a Yellow Card?	Yes =219 (38.4%); No =351 (61.1%)
L) Roughly how many Yellow Cards have you submitted?	1 = 77 (35.2%); 2 to 5 = 114 (52.1%); 6 to 10 = 13 (5.9%); 11 to 20 = 10 (4.6%); 21 to 30 = 3 (1.4%); >30 = 2 (0.9%)
M) What reason(s) prevent you from reporting to the Yellow Card Scheme? (please select all that apply)	Not applicable, I report every adverse drug reaction I identify =202; I have never seen an adverse drug reaction =261; I am not confident about identifying adverse drug reactions =26; I am not confident about what to report on a Yellow Card =26; I do not know where to access a Yellow Card =4; I do not have access to a Yellow Card when I am considering reporting =5; I often do not have all of the information needed to complete a report =32; Another member of my team reports so I do not feel I need to =12; I assume other healthcare professionals dealing with the patient will report, so there is no point sending a duplicate report =19; I have no time to report =21; Only see known ADRs =12; Other =10; Not applicable =38.
N) Have you ever received training on adverse drug reactions and reporting to the Yellow Card Scheme from anywhere other than your NMP course?	Yes =380 (66.7%); No =190 (33.3%)
O) Would you value additional information/support/training about adverse drug reactions and the Yellow Card Scheme from YCC North West to support your continued professional development (CPD)?	Yes =475 (83.3%); No =61 (10.7%); Not sure =34 (6.0%)

Of the 570 respondents, the majority were either nurses (68.1%) or community practitioners (eg district nurse and health visitor) (21.1%), with most completing their NMP training at institutes located in the north‐west of England (94.2%). The average duration between completion of professional qualification and NMP certification was 14 years (SD = 10 years; range =0‐39 years), with 77.2% gaining their NMP certification within the last 10 years (ie from 2007 onwards). The majority of NMPs had exercised their right to prescribe with only 16 (2.8%) indicating that they had not prescribed. Almost 50% of NMPs were prescribing at least once daily.

Not surprisingly, the majority of respondents (95%) had received training about the YCS, but this was delivered in different ways, with the majority (56%) stating that it was delivered as part of another session. Only a small proportion of the respondents (26.7%) stated that they were required to complete a Yellow Card as part of their training.

Only 219 of the respondents (38.4%) reported submitting a Yellow Card, with the majority of these individuals submitting five or less Yellow Cards; 28 (4.9%) stated they had submitted more than five Yellow cards. The reported profession had an impact on the likelihood of submitting to the YCS (*χ* ^2^ = 19.3, *P* < 0.0005), with community practitioners having the lowest proportion (28/120; 23.3%) and “other” professions having the highest proportion (34/62; 54.8%). Further exploration of the data revealed that pharmacists were generally responsible for this trend (26/27; 96.3%). Being professionally qualified for more years (*B *= 0.30, *P *< 0.0005; 95% CI 1.01 to 1.05) and receiving additional training support about ADRs and the YCS (*χ* ^2^ = 14.7, *P* < 0.0005) were also associated with an increased likelihood of submitting ADR reports to the YCS. Responses to questions C, D, E and I were not statistically associated with submitting to the YCS.

For those who answered “no” to “Have you ever reported an adverse drug reaction on a Yellow Card?” we explored reasons preventing reporting to the YCS (question M). Thirty‐eight (10.8%) individuals stated that reporting was not applicable. Although we cannot be certain, we believe that this is mainly because these professionals were not engaged with prescribing or patient contact in their current role, such as managerial/administrative positions. The most common reason given by all individuals (n = 261; 74.4%) who answered “no” to “Have you ever reported an adverse drug reaction on a Yellow Card?” was “I have never seen an adverse drug reaction.” Other reasons are summarized in Table 1.

We attempted to gauge an understanding of identification of ADRs via Question J (Table 2). The majority of respondents stated they were “confident” or “very confident” at identifying ADRs (pooled n = 432). Years qualified as an NMP did not impact confidence (*P *= 0.145), but self‐reported confidence in the detection and reporting of ADRs immediately after completion of NMP training was significantly associated with current confidence in identifying ADRs (*P *< 0.0005) (ie higher confidence after NMP training resulted in more confidence at the time of the survey). There was a positive linear relationship between confidence in identifying ADRs and likelihood of ever reporting to the YCS as evidenced by the proportion (as a percentage) of individuals submitting a YC. There was also a significant association between confidence and the number of YCs submitted (*P *< 0.0005), but this did not follow an absolute linear trend. Over 80% of the cohort indicated that they would value future additional information/support/training on ADRs and/or the YCS.

**Table 2:** Differences within ordered‐category answer patterns for question J (“How confident are you are at identifying adverse drug reactions?”)

	Very confident	Confident	Neither confident nor not confident	Not confident	*P*‐value
Respondents (n)	52	380	118	20	NA
Percentage of total respondents	9.1%	66.7%	20.7%	3.5%	NA
Mean time since completed NMP training (y)	6.0 ± 3.5	6.0 ± 4.6	7.3 ± 5.1	7.5 ± 7.1	0.145^b^
Averaged ordered‐category matching confidence immediately after NMP training (lower score =higher confidence)	1.10 ± 0.36	1.77 ± 0.62	2.43 ± 0.69	2.65 ± 0.93	<0.0005^c^
Percentage submitted YC to YCS	59.6%	41.3%	22.8%	20%	NA
Averaged ordered‐category matching mean ± SD number of YCs submitted to YCS (n = 219^a^)	2.39 ± 1.31	1.86 ± 0.83	1.41 ± 0.50	1.75 ± 1.5	<0.0005^c^

a Only those who reported submitting a Yellow Card to the YCS were included.

b Derived from Kruskal‐Wallis H test.

c Derived from Spearman's rank correlation coefficient.

### 3.2 Discussion

The aim of this questionnaire was to gain an understanding of the training received by NMPs on pharmacovigilance, their confidence in identifying ADRs and their engagement with the YCS. The questionnaire attracted 570 valid responses from various healthcare professionals. Although we cannot calculate the response rate as we do not know the true NMP denominator in the north‐west of England, this is one the largest studies investigating the perceptions of healthcare professionals to ADR reporting. This gives us confidence that the results are broadly representative but should still be interpreted with caution.

Most participants agreed that their NMP course had left them feeling “very confident” at being able to detect ADRs and a similar proportion reported they were confident or very confident at identifying ADRs at the time of completing the questionnaire, which is a positive outcome given the high rates. Indeed, those with self‐reported confidence in identifying ADRs were three times more likely to have submitted a Yellow Card. There was also a significant association between confidence and number of Yellow Cards submitted. However, when our results are taken as a whole, this confidence in identifying and reporting ADRs is inconsistent with the fact that only 38.4% of the respondents had submitted a Yellow Card. It is known that the majority of healthcare professionals (including medical prescribers) do not report ADRs: indeed, a systematic review of 37 papers demonstrated that the median underreporting rate of ADRs was 94% (interquartile range: 82%‐98%), with similar rates between medical prescribers and other healthcare professionals.^8^ Moreover, a survey of 280 hospital pharmacists reported that 46% had identified ADRs in the previous 6 months that should have been reported,^9^ but only a small proportion (15%) did so. Additionally, one previous study of NMP engagement with pharmacovigilance found that almost half of respondents had reported ADRs to the YCS, although the last submission made by an individual was often before their NMP training was completed.^7^

The design of our study is similar to that of Stewart and colleagues, and many of the outcomes complement these previous findings.^7^ A common theme between both studies is respondents’ lack of engagement with formal reporting despite high confidence and/or willingness to participate in pharmacovigilance. This disparity appears to be greater in the present study where the percentage of respondents reporting to the YCS was lower (38.4% vs 58.6%), although this might reflect the greater proportion of pharmacists in the Stewart study. Other common outcomes include NMPs not viewing time as a limiting factor in submitting YCs and desire for further training.

The reasons why healthcare professionals, both medical and nonmedical, do not report ADRs have been extensively studied through attitudinal surveys. The most common reasons reported include (a) a lack of time to report,^10^, ^11^ which may in fact demonstrate that ADR reporting is not a priority for most; (b) uncertainty regarding which drugs and/or reactions need to be reported^12^, ^13^; (c) fear that submitting a *suspected *ADR that is subsequently disproved may cause embarrassment^14^; (d) uncertainty whether a single report will make a contribution^15^; and (e) fears regarding litigation if they have administered a prescription that resulted in an ADR.^15^ There appears to be a difference in one attitudinal construct to reporting between medical prescribers and other healthcare professionals, in that medical prescribers are less likely to view reporting as another person's responsibility. Interestingly, primary care staff appear to be more likely to report than secondary care colleagues.^15^, ^16^ In our survey, pharmacists were most likely to have ever reported to the YCS. Although the number of pharmacists represented in our survey is small (n = 27), it is consistent with a previous study,^7^ perhaps reflecting the pharmacology training received by pharmacists alongside greater access to additional drug information as part of their daily workload. In our survey, the commonest reason for not reporting was that the respondent had never seen an ADR. This seems unlikely given the high prevalence of ADRs and probably indicates substantial uncertainty in identifying and recognizing ADRs in clinical practice.

Training is critical in improving pharmacovigilance practice in prescribers and is known to be a positive predictor for future reporting.^17^ In our survey, although almost all respondents had received training in this area, this was largely delivered as part of another session, with only a minority having had a dedicated session on ADR reporting. We would recommend that those who organize courses, and those who accredit them, ensure that there are sessions solely dedicated to the importance of ADRs in clinical practice and reporting of these ADRs. NMP courses not only have theoretical training but all NMPs are supposed to spend time with a designated medical professional to enhance theory through practical training in clinical settings. Perhaps as part of the time spent with a designated medical professional, all NMPs should be required to report an ADR on a Yellow Card. Indeed, 26.7% of our respondents were required to complete a Yellow Card as part of their training and, although not a significant predictor of future YC reporting in our study, there is absolutely no reason why this figure should not be 100%. Clearly, training in pharmacovigilance should not end at the time when the NMP qualifies to prescribe, but should continue throughout their professional life as part of continuing professional development (CPD). Indeed, in our survey, over 80% of individuals wanted further training.

Our survey is of course a snapshot of NMPs and has inherent limitations. First, we lack an accurate denominator for the population under investigation and therefore are unable to demonstrate whether our sample is representative. Furthermore, these data may not represent other areas of the UK and profession‐specific interpretations are limited due to small samples from certain cohorts. However, our sample is relatively large for this type of study, and a crude estimate shows we captured data from around 20% of the NMP population in the north‐west of England. It is clear that overall, there needs to be a certain level of caution when interpreting the findings. Second, we also cannot exclude the possibility that the responses of nonparticipants may have been different to the responses received nor can we exclude the risks of recall bias and social desirability bias. Third, there is a lack of data regarding the completeness and value of Yellow Cards submitted by NMPs. Although this is a wider technical issue outside the control of this study, it does limit the impact of the findings. Finally, it would have been informative to have a measure of the volume of prescribing rather than just frequency. Such data would have enabled analysis of the association between prescribing volume and reported outcomes.

## 4 WHAT IS NEW AND CONCLUSION

NMPs have an important role in drug safety and need to be encouraged to engage with formal pharmacovigilance systems such as the YCS. Training appears to give NMPs confidence to identify ADRs, but there seems to be a gap in actually identifying ADRs given that most reported that they had never seen an ADR. Strategies for improving the translation of theoretical knowledge about ADRs into practical skills in identifying ADRs, and subsequently reporting them, will be important. Minor modification of the current training courses to emphasize the importance of ADRs will be important in ensuring that future NMPs fully engage with spontaneous reporting systems such as the YCS. This survey has provided a baseline assessment to help direct future resource allocation in training and research provision. Further work is needed to add understanding to the gaps initially identified and subsequently explore interventions.

## CONFLICT OF INTEREST

The authors have stated explicitly that there are no conflict of interests in connection with this article.
